# Effects of Non-Opioid Analgesics on the Cell Membrane of Skin and Gastrointestinal Cancers

**DOI:** 10.3390/ijms23137096

**Published:** 2022-06-26

**Authors:** Natalia Janicka, Agnieszka Sałek, Magdalena Sawińska, Ernest Kuchar, Anna Wiela-Hojeńska, Katarzyna Karłowicz-Bodalska

**Affiliations:** 1 Faculty of Pharmacy, Wroclaw Medical University, 50-556 Wroclaw, Poland; n.janicka@student.umw.edu.pl (N.J.); agnieszka.salek@student.umw.edu.pl (A.S.); magdalena.sawinska@student.umw.edu.pl (M.S.); 2 Department of Pediatrics with Clinical Assessment Unit, Medical University of Warsaw, 02-091 Warsaw, Poland; ernest.kuchar@wum.edu.pl; 3 Department of Clinical Pharmacology, Faculty of Pharmacy, Wroclaw Medical University, 50-556 Wroclaw, Poland; anna.wiela-hojenska@umw.edu.pl; 4 Department of Drugs Form Technology, Faculty of Pharmacy, Wroclaw Medical University, 50-556 Wroclaw, Poland

**Keywords:** skin cancers, gastrointestinal cancers, non-opioid analgesics, membrane, modulation

## Abstract

Skin and gastrointestinal cancer cells are the target of research by many scientists due to the increasing morbidity and mortality rates around the world. New indications for drugs used in various conditions are being discovered. Non-opioid analgesics are worth noting as very popular, widely available, relatively cheap medications. They also have the ability to modulate the membrane components of tumor cells. The aim of this review is to analyze the impact of diclofenac, ibuprofen, naproxen, acetylsalicylic acid and paracetamol on skin and gastrointestinal cancers cell membrane. These drugs may affect the membrane through topical application, at the in vitro and in vivo level after oral or parenteral administration. They can lead to up- or downregulated expression of receptors, transporters and other molecules associated with plasma membrane. Medications may also alter the lipid bilayer composition of membrane, resulting in changes in its integrity and fluidity. Described modulations can cause the visualization of cancer cells, enhanced response of the immune system and the initiation of cell death. The outcome of this is inhibition of progression or reduction of tumor mass and supports chemotherapy. In conclusion, non-opioid analgesics may be used in the future as adjunctive therapy for the treatment of these cancers.

## 1. Introduction

Globally, cancer is very often diagnosed, and its mortality rate is still increasing. This is a severe problem for which there is no single therapeutic solution. Cancers of the skin and gastrointestinal tract are worth noting. The most frequently detected and best described skin cancers are melanoma, basal cell carcinoma (BCC) and squamous cell carcinoma (SCC). The incidence and mortality of skin cancer has been increasing over the years. The chemotherapy used does not always guarantee a cure due to the very rapidly progressive nature of the disease. Gastrointestinal cancers also have different prognoses and rates of progression depending on their location. In this review we concentrate mainly on colorectal and liver tumors. Patients often see a doctor when there are disturbing clinical symptoms, and the disease is already in an advanced stage. Late diagnosis reduces the effectiveness of therapy and the chance of recovery. Therefore, scientists are developing new compounds or testing well-known drugs for new indications and applications in cancer conditions including those at an advanced stage.

**Figure 1:**
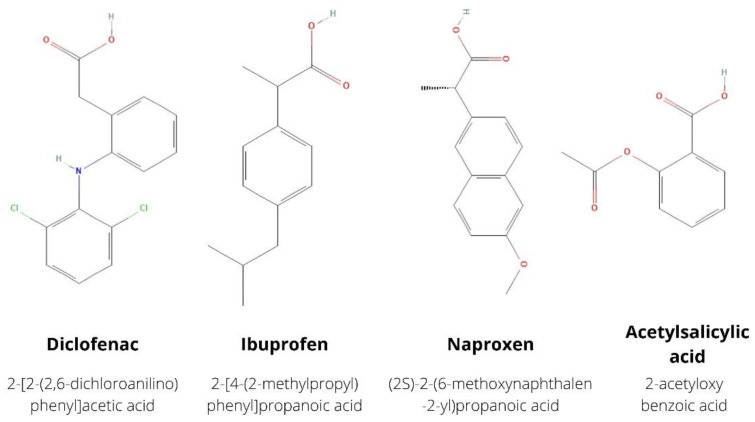
The structures of described in this review non-steroidal anti-inflammatory drugs (NSAIDs) with International Union of Pure and Applied Chemistry (IUPAC) name.

Non-opioid analgesics are those drugs by which analgesia can be achieved without affecting opioid receptors. These include nonsteroidal anti-inflammatory drugs (NSAIDs), acetaminophen, anticonvulsants (inhibitors of voltage-gated sodium or calcium ion channels, e.g., phenytoin, lamotrigine, carbamazepine, gabapentinoids, levetiracetam), antidepressants including tricyclic antidepressants (TCAs) and selective serotonin reuptake inhibitors (SSRIs) (e.g., amitriptyline, nortriptyline, duloxetine, venlafaxine), TRPV1 antagonists (capsaicin), cannabinoids, and muscle relaxants (e.g., benzodiazepines) [1]. The World Health Organization (WHO) has introduced an analgesic ladder to determine the degree of pain and appropriate pharmacotherapy in cancer pain and acute and chronic painful non-cancer disorders. Non-opioid analgesics such as NSAIDs or acetaminophen are the drugs of choice on the first step of this analgesic ladder for the treatment of mild pain and as supportive drugs on the higher steps [2,3]. They are among the most commonly used over-the-counter (OTC) medications. These drugs are taken for self-treatment of conditions with elevated temperature, colds, inflammation, and pain of various origins. In this paper we concentrate on selected NSAIDs (diclofenac, ibuprofen, naproxen and acetylsalicylic acid, which structures and names are shown in Figure 1) and acetaminophen. NSAIDs are the most commonly used medicines in the world [4]. They have analgesic and antipyretic effects and inhibit inflammation from taking place in the body. NSAIDs act as inhibitors of cyclooxygenase (COX), which determines the synthesis of prostaglandins. Prostanoids are associated with inflammation, infection, and cancer [5]. The effects of COX-2 overexpression observed especially in skin and gastrointestinal cancers also include increased adhesion to the extracellular matrix, higher BCL2 level (which promotes apoptosis), downregulated TGFβ_2_ receptor expression, and lack of E-cadherin protein. Arachidonic acid, which concentration is very high after NSAIDs treatment, stimulates the enzyme sphingomyelinase to convert sphingomyelin to ceramide. This change leads to apoptosis induction in colon cancer cells [6]. Cytokines, chemokines, and prostaglandins (PGs) participate in the chronic inflammation that can initiate and promote tumor progression. Tumor cells also secrete inflammatory mediators, which confirms the important role of PGs in carcinogenesis [7]. Studies note that COX-2 overexpression contributes to increased expression of vascular endothelial growth factor (VEGF), which is associated with angiogenesis and nutrient delivery to colorectal cancer cells for proliferation or invasion. In colorectal cancer invasion, PGE2 plays a significant role through PI3K or EGFR-mediated c-Met activation leading to loss of cell adhesion. COX-2 by affecting β1-integrins, activation of matrix metalloproteinases and adhesion can also promote metastasis [8]. Therefore, inhibition of inflammation by COX blockade may be a target for cancer prevention and adjuvant treatment. However, an interesting topic worth exploring is the modulation of tumor cell membrane components by NSAIDs. Paracetamol (acetaminophen) is the most popular non-prescription drug in the world. It cannot be classified as NSAID because it has no anti-inflammatory activity. The detailed mechanism of action of this drug is still unknown. Several theories are related to the inhibition of prostaglandin formation and COX-independent mechanisms [9]. Acetaminophen may also affect tumor cell membrane components such as Beclin-1 and TNF-α, which have therapeutic value. These drugs are increasingly used mainly for pain therapy, so it is worth considering their other properties as adjunctive treatment in cancer. Our review focuses on non-opioid analgesics and their potential use in skin and gastrointestinal cancers.

Many studies prove that NSAIDs have chemopreventive effects by preventing the development of skin or gastrointestinal cancers [10,11,12]. However, this overview aims to assess the impact of selected and widely available non-opioid analgesics on tumor cell membrane modulation and their potential indications. First, we overview the influence of topically applied diclofenac on the cell membrane in non-melanoma skin cancers and on melanoma cell lines. Next, we describe the role of naproxen and ibuprofen administered orally or parenterally in murine models and in vitro studies in regulating membrane antigen expression in skin cancers. In the following paragraph, we discuss the action of diclofenac, ibuprofen, acetylsalicylic acid, and acetaminophen concerning changes in membrane elements in selected gastrointestinal cancers cell lines. Promising results from research on non-opioid analgesics may significantly impact the effectiveness of therapy.

## 2. Non-Opioid Analgesics in Skin Cancers

Skin cancers can be divided into non-melanoma skin cancers (NMSCs) and melanoma. NMSCs include basal cell carcinoma (BCC) and squamous cell carcinoma (SCC). The morbidity of BCC and SCC exceeds the number of melanoma diagnoses. However, malignant melanoma accounts for up to 90% of deaths among skin cancer patients. The incidence of melanoma is also related to the increased exposure to sunlight and an active outdoor lifestyle that has developed over recent years [13,14]. In 2020 1,198,073 new cases of the NMSCs were reported worldwide, excluding BCC, representing 6.2% of all sites. The number of deaths of all NMSCs was 63,731. The morbidity of melanoma was estimated at 324,635 cases, while mortality was 57,043 [15]. Late diagnosed malignant lesions give patients a worse prognosis, which is connected with increased mortality. Therefore, the search is for substances that have a therapeutic effect and inhibit the growth and invasion of cancer cells. A significant contribution of COX-2 was observed in murine and human melanoma models [16,17]. This isoform is associated with melanoma progression and poor prognosis. For this reason, it seems reasonable to use COX inhibitors, which are commonly used and readily available [18]. As a consequence, it may reduce skin cancer development. Non-opioid analgesics applied topically, internally and in vitro studies may also affect proteins in the membrane of tumor cells in a mechanism unrelated to inhibition of COX, as described below.

### 2.1. Non-Melanoma Skin Cancers (NMSCs)

#### 2.1.1. Diclofenac

**Figure 2:**
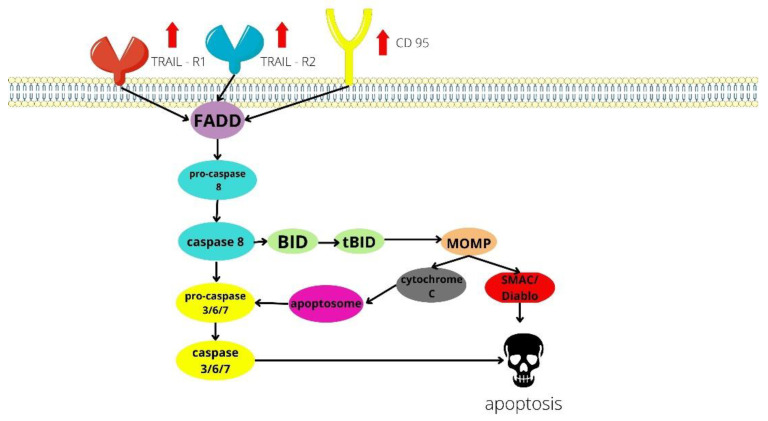
Diclofenacs impact on squamous cell carcinoma, including the cell membrane. Factor-related apoptosis-inducing ligand receptor 1 (TRAIL-R1), Factor-related apoptosis-inducing ligand receptor 2 (TRAIL-R2) and cluster of differentiation 95 (CD95), are showing greater membrane expression after diclofenac administration [24]. Both types lead to cell death in the same signalling pathway [29]. Interaction between receptors and their ligands leads to the recruitment of FADD. Pro-caspase 8 binds to FADD and becomes active caspase 8. Caspase 8 activates procaspases 3, 6 and 7 or leads to process BID (BH3 interacting-domain death agonist), releasing a truncated form of BID (tBID). tBID translocates to mitochondria and induces MOMP, which causes release of optogenic proteins (cytochrome C and SMAC/Diablo) from the mitochondria to the cytosol. Under the influence of cytochrome c, pro-caspase 9 and APAF-1 (Apoptotic protease activating factor-1) form the apoptosome. SMAC/Diablo inactivates members of IAPs (inhibitors of apoptosis), allowing proper caspase activation and apoptosis [30].

Topical application of diclofenac may be therapeutic when tumors are located in the upper layers of the skin [19]. Diclofenac sodium 3%, often in combination with hyaluronic acid 2.5% applied topically, is used in the treatment of actinic keratosis (AK), which is a precursor of invasive squamous cell carcinomas (iSCCs) [20,21]. Diclofenac in AK is applied twice daily for 2–3 months [19]. It can penetrate intact and diseased skin, which is a requirement for external application of the drug. After diclofenac use, the severity of side effects is less than after other topical drugs. Topical diclofenac therapy is generally well tolerated by patients and side effects are most often limited to skin irritation [21,22]. Topical diclofenac can also be used BCC and SCC. In the 3 disorders discussed, the mechanism is related to inhibition of COX-2 and production of PGE2, which is overexpressed in unhealthy cells [22,23]. In studies on four cell lines: SCL-I, SCL-II, SCC-12, and SCC-13 derived from facial SCC lesions, diclofenac in the form of 0.3% diclofenac/hyaluronic acid up-regulates the expression of TRAIL-R1 (factor-related apoptosis-inducing ligand receptor 1), TRAIL-R2 (factor-related apoptosis-inducing ligand receptor 2), and CD95 receptors on melanoma cells, which contributes to ligand death-induced apoptosis. These modulations are shown in Figure 2. This NSAID’s down-regulation of c-FLIP (antiapoptotic factors) also leads to programmed death [24,25]. These modulations may reduce skin cancer development. An in vitro study of a hybrid for AK and SCC topical treatment is underway. The hybrid is obtained by combining diclofenac with natural molecules characterized by antioxidant and antiproliferative activities [26]. This may increase therapeutic efficacy in the future.

Studies in vivo performed in Skh-1 hairless mice, proved that after topical application of 500 µg diclofenac for 15 weeks, tumor mass in SCC was reduced [27]. Phase II randomized controlled trial suggests that topical application twice daily under occlusion of diclofenac sodium 3% gel in hyaluronic acid 2.5% shows a promising effect in superficial basal cell carcinoma (sBCC) instead of calcitriol. Outcomes were determined by Ki-67 and Bcl-2 expression in tumor cells after treatment. More than 90% of patients experienced no adverse events and if they did occur, they were classified as mild to moderate. Most commonly reported were erythema, pruritus, swelling, and wounds at the target tumor site. Therapy was stopped for 8 study participants due to the severity of side effects. Serious adverse events requiring hospitalization were noted for 3 patients, but the cause was not medications [28].

#### 2.1.2. Naproxen

Naproxen after parenteral (intraperitoneal) administration (1 mg in 100 μL PBS) decreases expression of proliferating cell nuclear antigen (PCNA) and cyclin D1. The effect inhibits the proliferation of UVB-induced SCC and BCC in Ptch1^+/−^/SKH-1 hairless mice [31]. It is worth noting that the presence of PCNA was detected by researchers in the nucleus and the cell membrane of tumors [32]. PCNA is significantly expressed in basaloid squamous cell carcinomas (BSCCs) [33]. Membrane-associated cyclin D increases the tumor cell’s susceptibility to invasion [34].

Naproxen, in the same in vivo murine model, also decreased the expression of N-cadherin, which as a transmembrane protein, is a marker of the epithelial-mesenchymal transition (EMT) process. However, the expression of E-cadherin increased [31]. This non-opioid analgesic intensifies apoptosis and inhibits tumor growth, which may have therapeutic implications.

### 2.2. Melanoma

#### 2.2.1. Diclofenac

**Figure 3:**
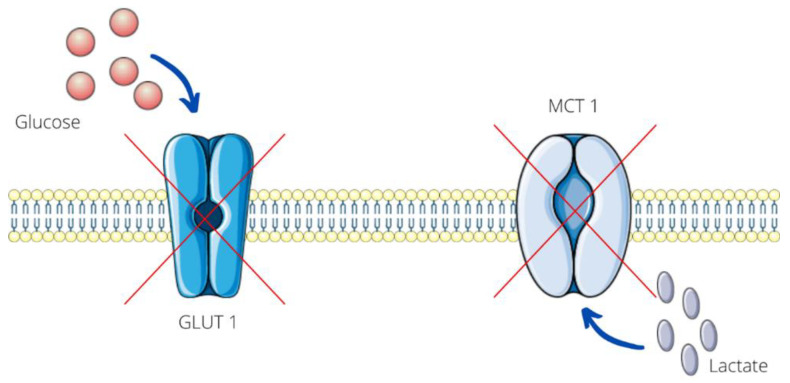
Diclofenacs impact on melanoma cellular transport. Glucose transporter 1 (GLUT1), monocarboxylate transporter 1 (MCT1) [35].

An in vitro study on the human melanoma cell line MelIm demonstrates that diclofenac at concentrations from 0.1 to 0.8 mM after 24 h and 48 h of incubation markedly reduces the expression of the transcription factor MYC, monocarboxylate transporter 1 (MCT1), and glucose transporter 1 (GLUT1)—the transmembrane protein responsible for the facilitated diffusion of glucose across a membrane. The effect of diclofenac on cellular transport is shown in Figure 3. Increased glycolysis is characteristic of cancer. Thus, this drug indirectly affects glucose metabolism by reducing glucose uptake and inhibiting glycolysis. This leads to decreased tumor growth [35]. MCT1 is responsible for the transfer of lactate across the membrane of cancer cells. Some researchers compare lactate to metabolic fuel [36]. This NSAID blocks the activity of not only MCT1 but also MCT4 in a non-COX-related mechanism. A carboxyl group plays a very important role in suppressing these transporters [37]. Diclofenac inhibiting lactate secretion from melanoma cells leads to the accumulation of this substance. There is a connection between intracellular lactate levels and poorer prognosis in patients [38]. Therefore, it can be concluded that diclofenac has an antiproliferative effect [39]. Moreover, diclofenac may be a promising solution in treating melanoma skin metastases [19]. This drug at concentrations of 0.1 and 0.2 mM after 72 h mildly increases the expression of major histocompatibility complex class I (MHC-I) molecules in M579 cell line [37]. MHC-I is expressed on the surface of melanoma cells and is involved in antigen presentation to CD8 + cytotoxic T lymphocytes (CTL). Recognition of a foreign molecule by CTL results in lysis of tumor cells. Thus, an up-regulation of MHC-I expression contributes to the visualization of melanoma cells and enhances the host immune response. This promotes the elimination of cancer cells and may limit tumor growth [40].

#### 2.2.2. Naproxen

Nitric oxide (NO•)-releasing nonsteroidal anti-inflammatory drugs (NONO-NSAIDs) as an innovative combination is the subject of research. One of these drugs is NONO-naproxen, which at a concentration of 0.1 mM, decreases surface activation of β1 integrin on human melanoma M624 cells after one hour of incubation [41]. The combination of β1 and α4 subunits(very late antigen-4 VLA-4, integrin α4β1) is a receptor for vascular cellular adhesion molecule-1 (VCAM-1) [42]. Downregulation of integrin activity leads to diminished melanoma cell adhesion to endothelial VCAM-1. Naproxen does not affect the adhesion of tumor cells to VCAM-1 or fibronectin compared to NONO-naproxen, which reduces metastasis. Released the diazeniumdiolate (NO•)-donor moiety plays a significant role in this mechanism of action [43]. Therefore, this class of non-opioid analgesics may be used in suppressing tumor expansion.

#### 2.2.3. Ibuprofen

An in vitro study on the BRAF^V600E^ A375 and the NRAS^Q61R^ SK-MEL-2 melanoma cell lines confirms that COX-2 expression positively modulates the expression of programmed death-ligand 1 (PD-L1)-trans-membrane protein found on the antigen presenting cells (APCs) as well as tumor cells. This ligand binds with PD-1 receptor on T-cells and blocks its activation. It inhibits the body’s immune response by avoiding the cytolysis of cancer cells. Consequently, downregulation of COX-2 and PD-L1 expression by NSAIDs potentiates the effect of cytotoxic T lymphocytes (CTL) in melanoma cell lines [44,45]. Thus, NSAIDs can increase the visualization of tumor cells to the immune system, which is tasked with killing foreign cells. In vivo studies in murine models of melanoma show that ibuprofen acts synergistically with anti-PD-1 (aPD1) therapy after oral administration. Ibuprofen dissolved in drinking water at a concentration of 1 mg/mL was administered to 6–7 week old male C57BL6/j mice after prior implantation of YUMMER (Yale University Mouse Melanoma Exposed to Radiation) 1.7 cells. Taking into consideration the average water intake of these laboratory animals, the dose in humans would be equivalent to 1200 mg/day, reflecting the usual anti-inflammatory dose. Tumors regressed, their volume decreased, and survival increased compared to aPD1 monotherapy [46]. Therefore, a dose of ibuprofen that inhibits inflammation could also be an anticancer dose, which requires further study. This combination therapy may safely potentiate the therapeutic effect in humans in the future.

Ibuprofen (0.3 mM), an activator of peroxisomal proliferator-activated receptor α (PPARα), enhances CD36 expression in the human amelanotic melanoma cell line C32 [47]. CD36 (cluster of differentiation 95), a membrane glycoprotein, binds, for example, to long-chain fatty acids, phospholipids, and anti-angiogenic thrombospondin-1 (TSP-1) [48]. Increased expression of CD36 is associated with cancer metastasis and poor prognosis for patients, as confirmed by studies [49,50]. This glycoprotein affects angiogenesis and vasculogenic mimicry (VM) formation in melanoma. Both formed vessels deliver oxygen and nutrients to tumor cells, but TSP-1 does not inhibit VM formation compared to angiogenesis. CD36 interacting with integrin-α 3 and laminin plays a significant role in tumor adhesion [48]. Fatty acid (FA) uptake by CD36 is connected with increased levels of lipid signaling that may influence metastasis formation [51]. The result is increased melanoma progression, which is an unfavorable prognostic factor.

**Figure 4:**
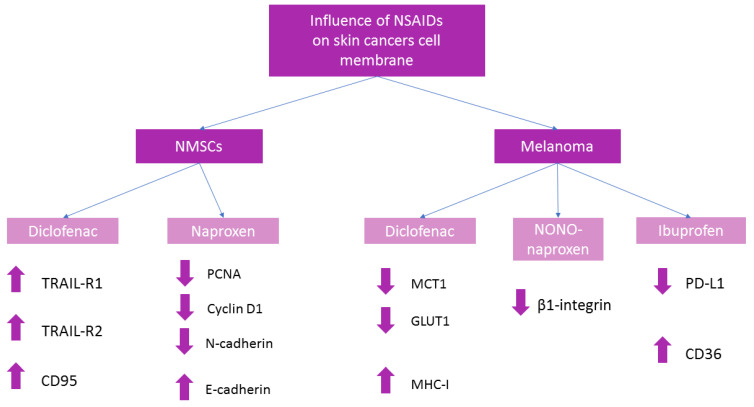
Summary of non-opioid analgesics influence on the melanoma plasma membrane. Non-melanoma skin cancers (NMSCs), nitric oxide-releasing naproxen (NONO-naproxen), factor-related apoptosis-inducing ligand receptor 1 (TRAIL-R1), factor-related apoptosis-inducing ligand receptor 2 (TRAIL-R2), cluster of differentiation 95 (CD95) [24], proliferating cell nuclear antigen (PCNA) [31], monocarboxylate transporter 1 (MCT1), glucose transporter 1 (GLUT1) [35], major histocompatibility complex class I (MHC-I) [37], programmed death-ligand 1 (PD-L1) [44,45], cluster of differentiation 36 (CD-36) [47].

The effects of non-opioid analgesics on the expression and activity of membrane components are summarized in Figure 4.

## 3. Non-Opioid Analgesics in Gastrointestinal Cancers

Gastrointestinal (GI) cancers are a severe problem on a global scale. In 2020, 6,577,800 new cases of GI cancers were reported worldwide. The most common tumor location was the colon, with 1,148,500 novel incidents [52]. Due to the high incidence, the possible impact of cheap and readily available drugs on the membranes of neoplastic cells is of interest. Non-opioid analgesics are widely used and well known, so we have extensive data on their safety and efficient distribution. Research shows that some of them have an effect on the membranes of cancer cells in the digestive system.

### 3.1. Diclofenac

Diclofenac shows an intriguing effect on colorectal cancer cells in vitro. The phenotype of these cells is dependent on COX-2 expression. In the cell line overexpressing COX-2 (HT29), the expression of intercellular adhesion molecule -1 (ICAM-1) and CD44 on a cell membrane was higher. In in vitro test, diclofenac in concentration of 0.42 mM, reversed the effect of COX-2 overexpression after 24 h incubation [53]. The analysis of samples taken from 74 cancer patients showed that, a lower CD44 level can improve the prognosis of patients with advanced colorectal cancer [54]. CD44 is a marker of poorly differentiated cells, correlated with higher malignancy [55]. On the other hand, the ICAM-1 presence is associated with a lower risk of metastasis and higher TILs infiltration [56]. For this reason, it cannot be conclusively said that the use of diclofenac in COX-2 expressing colorectal cancer is beneficial.

A different mechanism of action of diclofenac on HT-29 cells relates to the induction of oxidative stress. It was also observed in vitro [57]. Oxidative stress leads to the formation of reactive oxygen species (ROS). ROS can damage membrane lipids, both in cancer and healthy cells, which leads to loss of membrane properties [58,59]. Peroxidative damage leads to changes in membrane fluidity. It can also disturb the integrity of membrane proteins or cholesterol. Highly oxidizable lipids may attack more proteins and lead to an unfavorable excess of protein carbonyls [60]. Diclofenac significantly affects the membranes of HT-29 cells. Its use, however, requires consideration of the risk-benefit balance.

### 3.2. Ibuprofen

**Figure 5:**
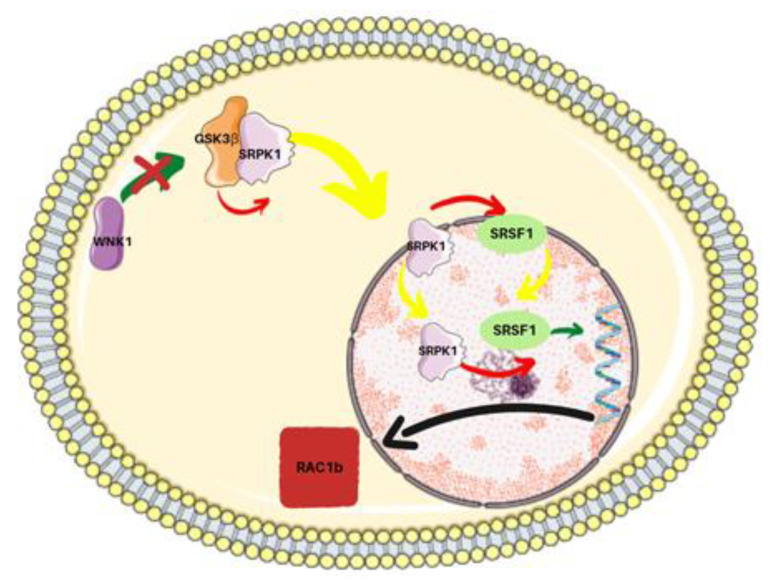
Mechanism of alternative splicing of RAC1b inhibition by ibuprofen. The red color of the arrow means phosphorylation, yellow translocation, and green binding. The symbol “x” indicates the site of action of ibuprofen. Lysine deficient protein kinase 1 (WNK 1), Glycogen synthase kinase 3 beta (GSK3β), Serine/arginine-protein kinase (SRPK 1), Serine/arginine-rich splicing factor 1 (SRSF 1), Ras-related C3 botulinum toxin substrate 1B (RAC1b) [62].

Ibuprofen seemed beneficial in colorectal cancer therapy because of its anti-inflammatory effect. Further research in vitro on a human collorectal adenocarcinoma cell line HT-29 showed its COX independent therapeutic mechanism consisting of the inhibition of expression RAC1b protein. The cell line HT29 was incubated for 48 h with a 500 µM of ibuprofen [61]. RAC1b is an isoform of the monomeric GTPase Rac1, obtained by alternative splicing and located mainly in the plasma membrane. Its presence increases the malignancy of the tumor and makes cells resistant to apoptosis [62,63]. In HT29 cells expressing RAC1B, alternative splicing occurs due to the phosphorylation of the splicing factor SRSF1 by SRPK1. SRPK1 forms a complex of cytoplasmic proteins together with WNK1 and GSK3β. Ibuprofen disrupts the interaction between WNK1 and GSK3β, leads to AKT1 phosphorylation of GSK3β, and thus prevents GSK3β phosphorylation of SRPK1. In this way, alternative splicing is blocked, which reduces RAC1B expression [61]. The mechanism of this action is shown in Figure 5. In the presence of ibuprofen, the viability of HT29 cells was reduced by 50% [64].

In human gastric adenocarcinoma (AGS) cell line, treatment with ibuprofen in concentration of 500 µM reduced the expression of the markers OCT4 and CD44. That could mean an increase in dedifferentiation of the cells or/and a reduction of the cancer stem cells [65]. Such a change may improve a patient’s prognosis.

### 3.3. Acetylsalicylic Acid

Hep3B and HepG2 cells are the subject of research in terms of oncosis induction by acetylsalicylic acid (ASA). The treatment of liver cancer cell lines with ASA concentrations from 8 to 16 mM for 48 h increased the dose-dependent cytotoxicity. The progress of cell death was investigated through the use of imaging microscopy. In Hep3B and HepG2 cells, cytoplasmic edema and cell membrane vesicles were noticeable [66]. Dose- and time-dependent inhibition of proliferation is also seen in the following study, where the liver cancer cell lines Huh-7, Hep-G2, Hep-3B, Li-7, HLE, HLF, and PLC/PRF/5 were investigated. A cell proliferation test was performed after 48 h of exposure to the following ASA concentrations: 2.5.5 or 10 mmol/L; and the results showed significant inhibition of proliferation. The matrix system to measure p-EGFR levels following acetylsalicylic acid treatment indicated inhibition of the receptor expression. EGFR is an epidermal growth factor receptor belonging to the family of receptor tyrosine kinases. EGFR is located in the cell membrane during the absence of stimulation, and its frequent similarity to mutations and overexpression has been observed [67]. In summary, the increased level of miR-137 (non-coding RNA molecule) resulted in inhibition of EGFR expression in the cell membrane, thus reducing the cell proliferation capacity [68].

Interesting results were obtained by researchers studying the effect of ASA on HCT116 colon cancer cell lines. Flow cytometric analysis showed a significant decrease in the expression of Fas ligand (FasL) for the Fas receptor (FasR) considered as the cell death receptor, compared to the control sample [69]. The transmembrane membrane protein FasL, belonging to the tumor necrosis factor (TNF) family, is known as the pro-apoptotic factor. It is composed of two intracellular and extracellular regions that take part in signaling pathways, for example, by stimulating receptors on T lymphocytes and being a ligand for the receptors [70].

### 3.4. Acetaminophen (Paracetamol)

Paracetamol is one of the most commonly used painkillers, especially for people who cannot take NSAIDs. The mechanism of action of acetaminophen is not fully understood, but it is believed that by acting on TRPV1 and /or CB1 receptors via the metabolite AM404, it causes analgesia [71].

In impact studies, IL-33 to autophagy and the innate immune response of hepatocytes in the context of in vivo and in vitro exposure to acetaminophen inter alia HepaRG, and Huh7 cell lines were used. Increased Beclin-1 expression has been noted as a result of the action of acetaminophen on liver cancer cell lines [72]. Beclin-1 was observed in the plasma membrane of cancer cells by immunostaining, but no such effect was found in healthy cells [73]. In a study with HepG2 cell carcinoma cell lines by ELISA, an increase in TNF-α levels was found after acetaminophen exposure [74]. TNF-α as a tmTNF-α transmembrane protein in the form of stable homodimers, binding to its receptors TNFR1 and TNFR2, plays a role in the transmission of molecular signals such as inflammation and cell death [75,76].

Oxidative stress caused by acetaminophen can also affect the integrity and function of the cell membrane. Among other things, the integrity of the membrane of cells from the HepG2 cell line was checked in the following study. LDH test was performed after acetaminophen treatment, resulting in a significant LDH release increase from 21.73 ± 5.94% to 46.86 ± 2.81%. Incubation of HepG2 cells with 20 mM acetaminophen resulted in a significant (4.95-fold) increase in lipid peroxidation, as demonstrated by the peroxidation test. The lipid peroxidation test as an indicator of lipid oxidative damage using malondialdehyde is based on the intensity of the color change caused by the reaction of MDA with thiobarbituric acid (TBA) [77]. Lipid peroxidation, and especially its products, have a significant influence on the modification of the physical properties of cell membranes. The changes that can be noticed in this process are an increase in the permeability for polar substances, especially for hydrogen ions, and a reduction in the potential difference on both sides of the lipid bilayer. In addition, increased induction of COX-2 expression was observed, as well as inhibition of the activity of some membrane enzymes and transport proteins, which consequently leads to the loss of plasma membrane integrity [78].

**Figure 6:**
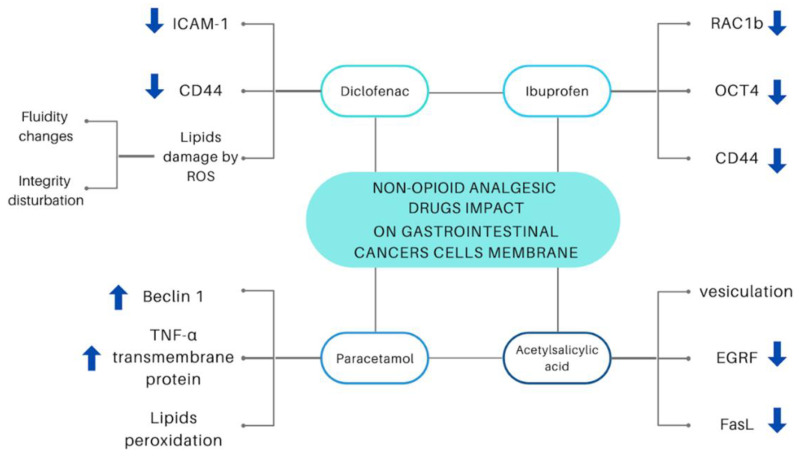
Changes in the lipid bilayer, including protein expression, caused by non-opioid analgesic drugs. Intracellular Adhesion Molecule-1 (ICAM-1) [53,56], antigen on a cell surface (CD44) [53,54,55,65], Reactive Oxygen Species (ROS) [57,58,60], Ras-related C3 botulinum toxin substrate 1B (RAC1b) [61,62,63,64], Octamer-binding transcription factor 4 (OCT40) [65], Tumor necrosis factor α (TNF-α) [74,75,76], epidermal growth factor receptor (EGRF) [67,68], Fas ligand (FasL) [69,70], Beclin-1 protein [72,73].

Figure 6 shows the impact of discussed drugs on gastrointestinal cancers cells membranes.

## 4. Conclusions

Modification of cell membrane elements by non-opioid analgesics in the skin and gastrointestinal cancers is an interesting aim of anticancer therapy. Changes in the lipid bilayer and expression of surface molecules direct cancer cells to the death pathway and suppress proliferation and expansion. It contributes to the inhibition of growth and metastasis of tumor cells. The effect is mainly better therapeutic outcomes, which is relevant in oncology. However, relatively few studies identify the impact of non-opioid analgesics on cancer cell’s plasma membrane and explain the mechanism of this. The results so far are promising, which may encourage scientists further to explore the properties of this popular group of drugs and discover new indications for other cancers as well. Non-opioid analgesics can hopefully be used as adjunctive treatment in the future.
